# Redox Regulation of Calcium Signaling in Cancer Cells by Ascorbic Acid Involving the Mitochondrial Electron Transport Chain

**DOI:** 10.1155/2012/921653

**Published:** 2012-11-25

**Authors:** Grigory G. Martinovich, Elena N. Golubeva, Irina V. Martinovich, Sergey N. Cherenkevich

**Affiliations:** Department of Biophysics, Belarusian State University, Nezavisimosti Avenue 4, 220030 Minsk, Belarus

## Abstract

Previously, we have reported that ascorbic acid regulates calcium signaling in human larynx carcinoma HEp-2 cells. To evaluate the precise mechanism of Ca^2+^ release by ascorbic acid, the effects of specific inhibitors of the electron transport chain components on mitochondrial reactive oxygen species (ROS) production and Ca^2+^ mobilization in HEp-2 cells were investigated. It was revealed that the mitochondrial complex III inhibitor (antimycin A) amplifies ascorbate-induced Ca^2+^ release from intracellular stores. The mitochondrial complex I inhibitor (rotenone) decreases Ca^2+^ release from intracellular stores in HEp-2 cells caused by ascorbic acid and antimycin A. In the presence of rotenone, antimycin A stimulates ROS production by mitochondria. Ascorbate-induced Ca^2+^ release in HEp-2 cells is shown to be unaffected by catalase. The results obtained suggest that Ca^2+^ release in HEp-2 cells caused by ascorbic acid is associated with induced mitochondrial ROS production. The data obtained are in line with the concept of redox signaling that explains oxidant action by compartmentalization of ROS production and oxidant targets.

## 1. Introduction

Redox processes involving transfer of electrons or hydrogen atoms are central processes of energy conversion in respiratory organisms. Recently, it has become apparent that numerous functionally significant biological processes proceed with participation of physical mechanisms ensuring intermolecular electron transfer. Electron transfer between low-molecular weight components of cytosol and intracellular proteins leads to the change of a functional state of both cellular proteins and cells as a whole [1, 2]. All biological systems contain redox elements that play an important role in transcriptional regulation, cell proliferation, apoptosis, hormonal signaling, and other fundamental cell functions [3]. Organization and coordination of the redox activity of these elements occur through redox circuits and depend on the intracellular concentration of redox-active molecules [4, 5]. Redox active molecules may cause both regulatory and toxic effects depending on the value of cellular redox state parameters [5, 6]. However, little is known about mechanisms of regulation, structural organization, and interaction between electron-transport participants inside the cell and other signal and regulatory systems.

Recently new effects of such a redox-active molecule as ascorbic acid have been found. Beside numerous regulatory properties (hydroxylation of collagen, biosynthesis of carnitine and noradrenaline, etc.), selective cytotoxicity of high concentrations of ascorbic acid towards cancer cells has been described. Ascorbic acid in concentrations of 1–10 mM was shown to induce the death of prostate cancer cells, stomach cancer cells, and acute myeloid leukemia cells [7, 8]. In experiments* in vitro*, ascorbate cytotoxicity (EC_50_ < 4 mM) was observed in many types of cancer cell lines, whereas normal cells were resistant [9, 10]. Ascorbic acid treatment in high pharmacological concentrations significantly impeded tumor progression* in vivo* without toxicity to normal tissues [11, 12]. Thus, ascorbic acid at high doses possesses anticancer properties, but mechanisms of its selective cytotoxicity and targets of its action are still obscure.

It was shown that in the presence of transition metal ions and ascorbic acid, H_2_O_2_ is formed [13]. These data suggest that ascorbate cytotoxicity may be due to its ability to generate H_2_O_2_ [7, 12]. But this hypothesis does not appear to be compatible with facts. Recent studies have indicated that the intravenous injection of ascorbic acid in high (up to 8 mM) concentrations was not accompanied by the formation of H_2_O_2_ in blood [14]. It was also shown that even in the presence of transition metal ions and H_2_O_2_, ascorbate acted as an antioxidant that prevented lipid peroxidation in human plasma *in vitro* [15]. Previously, we have found that ascorbic acid regulates calcium signaling in human larynx carcinoma cells [16]. Ascorbate at concentrations in the range 3–10 mM activated cytosol pH value decrease and Ca^2+^ release from thapsigargin-sensitive intracellular Ca^2+^ stores. Although the ability of ascorbic acid to induce Ca^2+^ mobilization is shown, the precise mechanisms involved in Ca^2+^ release are not known. We proposed that mitochondria can be involved in ascorbate-induced calcium signaling. The aim of this study was to explore the participation of mitochondrial enzymes in regulation of calcium signaling in human larynx carcinoma HEp-2 cells by ascorbic acid.

## 2. Methods and Materials

### 2.1. Cell Culture and Reagents

Ascorbic acid was obtained from Himhrom Ltd. (Minsk, Belarus). Dulbecco's modified Eagle's medium (DMEM), fura-2-acetoxymethyl (AM) ester, 2′,7′-dichlorodihydrofluorescein diacetate (H_2_DCF-DA), rotenone, antimycin A, catalase, and HEPES were purchased from Sigma-Aldrich (St. Louis, MO, USA). Human larynx carcinoma HEp-2 cells were purchased from the Republican Research and Practical Center for Epidemiology and Microbiology (Minsk, Belarus). The cells were cultured at 37°C under a humidified atmosphere with 5% CO_2_ in DMEM supplemented with 10% fetal calf serum, 2 mM glutamine, and 80 mg/mL gentamicin. Rotenone (50–150 *μ*M), antimycin A (5–40 *μ*M), ascorbate (3–10 mM), and catalase (500 U/mL) were used. 

### 2.2. Fluorescent Spectrofluorimetry

Measurements of the free calcium ions' cytosol concentration ([Ca^2+^]_cyt_) were performed as previously described [17]. HEp-2 cells were loaded with 2.5 *μ*M fura-2 AM, washed, and mounted under continuous stirring in the chamber of the spectrofluorimeter (LSF 1211A, Minsk, Belarus). The standard recording medium (KRH) contained 131 mM NaCl, 5 mM KCl, 1.3 mM CaCl_2_, 1.3 mM MgSO_4_, 6 mM glucose, and 20 mM HEPES, pH 7.4 (NaOH).

Intracellular ROS generation was recorded using H_2_DCF-DA, which is a nonpolar compound that is converted into a nonfluorescent polar derivative (H_2_DCF) by cellular esterases after incorporation into cells. Membrane-impermeable H_2_DCF is rapidly oxidized to highly fluorescent 2′,7′-dichlorofluorescein (DCF) in the presence of intracellular ROS [18]. After loading with 10 *μ*M H_2_DCF-DA for 30 min, HEp-2 cells were rinsed two times with KRH and DCF fluorescence intensity was measured using 488 nm excitation/530 nm emission settings. All experiments were carried out at 37°C.

### 2.3. Statistics

The data were expressed as means ± standard error of the mean (SEM). Statistical significances between means were assayed using Student's *t*-test. The values were taken as significantly different when *P* < 0.05.

## 3. Results and Discussion

**Figure 1:**
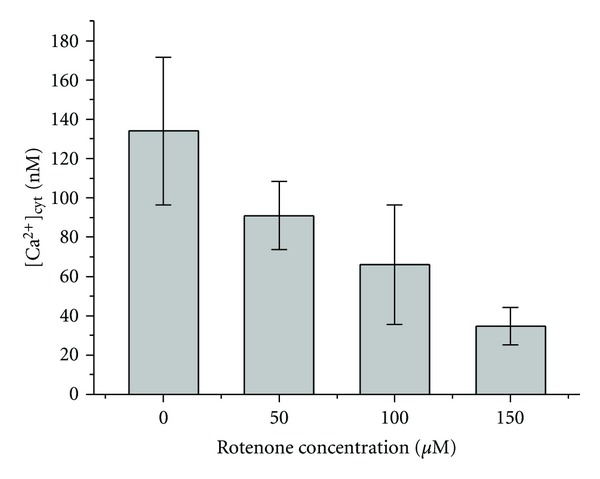
Influence of rotenone on ascorbate-induced increase of [Ca^2+^]_cyt_ in HEp-2 cells. Concentration of ascorbic acid in KRH—5 mM. Number of cells in 1 mL—2.5 × 10^6^.

In a previous study, ascorbic acid was found to cause a transient increase in [Ca^2+^]_cyt_ in human larynx carcinoma HEp-2 cells accompanied by depletion of intracellular thapsigargin-sensitive calcium stores [16]. Thapsigargin is known to induce Ca^2+^ release, from mitochondria and inositol-1,4,5-triphosphate-responsive Ca^2+^ stores. To examine involvement of mitochondria in ascorbate-induced Ca^2+^ release we used rotenone, mitochondrial complex I inhibitor. It was shown that treatment of cells with rotenone led to the decrease of ascorbate-induced Ca^2+^ release in HEp-2 cells (Figure 1), indicating that participation of mitochondrial enzymes in ascorbate induces Ca^2+^ release. There is no evidence that any specific receptors for ascorbate exist in mitochondria. To explain participation of mitochondria in ascorbate-induced Ca^2+^ release, we proposed a mechanism according to which an increase in ascorbate concentration could intensify ROS production by mitochondrial enzymes.

It is generally accepted that superoxide anion (O_2_^∙−^) is the primary free radical in mitochondria, which is formed as the result of electron “leak” from the electron transport chain elements to oxygen [19]. Ubisemiquinones generated in the respiratory chain were identified as possible donors of electrons for oxygen [20]. Here, it is reasonable to mention that ascorbate can reduce c-type cytochromes and b-type cytochromes [21, 22]. The evidence, taken together, suggests that electron transfer from ascorbic acid to cytochrome c_1_ or cytochrome c should decrease electron flow from ubiquinol to Rieske iron-sulfur cluster resulting in a rise of ubisemiquinone concentration and superoxide production. Dismutation of O_2_^∙−^ by the mitochondrial matrix Mn-superoxide dismutase leads to the formation of H_2_O_2_ [20]. H_2_O_2_, in turn, can oxidize thiol groups of targets and regulate Ca^2+^ release [23, 24]. To exclude possible generation of H_2_O_2_ by ascorbate in the extracellular solution catalase was used. Ascorbate-induced Ca^2+^ release from mitochondria was shown to be unaffected by catalase in an concentration of 500 U/mL (not shown). Thus, the important point in our model is that ROS production by ascorbic acid proceeds nearby specific redox sensors.

**Figure 2:**
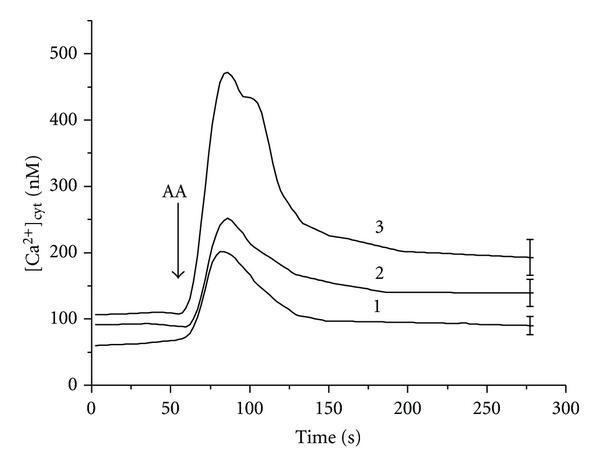
Influence of antimycin A on the ascorbate-induced Ca^2+^ release from HEp-2 cells mitochondria. Concentrations in KRH: ascorbic acid—5 mM; antimycin A (*μ*M): 1—0, 2—10, 3—15. Number of cells in 1 mL—3 × 10^6^. The arrow shows the instant of ascorbate addition.

**Figure 3:**
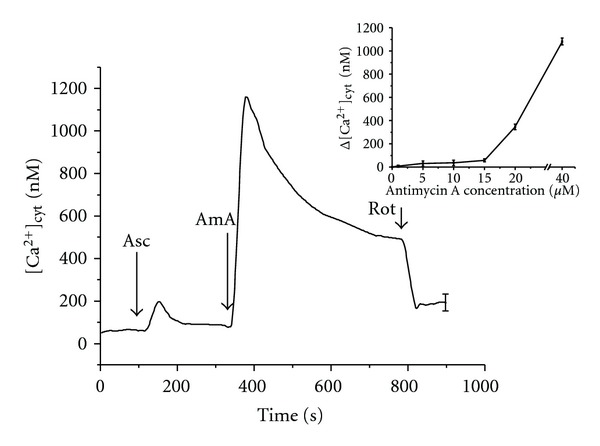
Influence of ascorbic acid and rotenone on the Ca^2+^ release from HEp-2 cells mitochondria under the antimycin A treatment. Concentrations in KRH: ascorbic acid—5 mM, antimycin A—10 *μ*M, rotenone—50 *μ*M. Number of cells in 1 mL—3 × 10^6^. Arrows show the instants of antimycin A (AmA), ascorbic acid (Asc), and rotenone (Rot) addition. The inset shows dependence of increase in [Ca^2+^]_cyt_ on the antimycin A concentration.

According to the proposed model, the increase of ROS generation in mitochondria is thought to induce Ca^2+^ release. The impact of this mechanism was assessed by using mitochondrial complex inhibitors antimycin A and rotenone. The increase in [Ca^2+^]_cyt_ in HEp-2 cells was detected after the application of mitochondrial complex III inhibitor antimycin A in concentrations above 20 *μ*M (Figure 3, inset). On the other hand, the increase in the amplitude and duration of ascorbate-induced Ca^2+^ release was observed even at lower concentrations of antimycin A (10 *μ*M) used in the study (Figure 2). Moreover, after the addition of ascorbate to HEp-2 cell suspension, antimycin A in this concentration induced a significant increase in [Ca^2+^]_cyt_. Subsequent addition of mitochondrial complex I inhibitor rotenone resulted in the decrease of [Ca^2+^]_cyt_ in cancer HEp-2 cells (Figure 3). These findings support our suggestion that ascorbate-induced Ca^2+^ release from HEp-2 cells mitochondria proceeds with the participation of ROS produced in the electron transport chain. 

**Figure 4:**
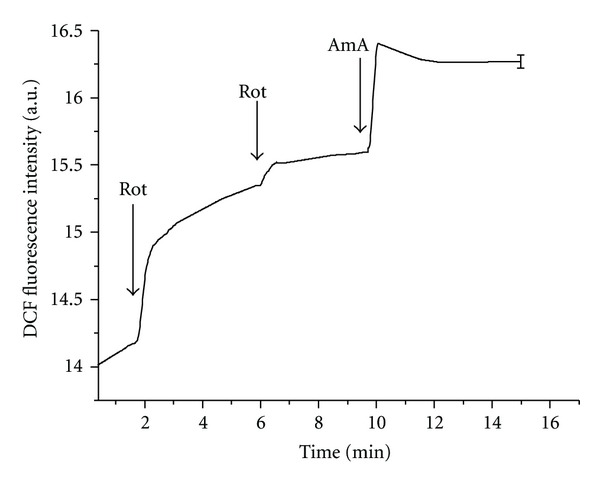
DCF fluorescence intensity in HEp-2 cells. Concentrations in KRH: rotenone—50 *μ*M, antimycin A—10 *μ*M. Number of cells in 1 mL—3 × 10^6^. Arrows show the instants of antimycin A (AmA) and rotenone (Rot) addition.

The major sites of superoxide formation within the mitochondrial respiratory chain are linked to NADH: ubiquinone oxidoreductase (complex I) and ubiquinol: cytochrome c oxidoreductase (complex III) [19]. In our experiments both rotenone and antimycin A were ascertained to enhance ROS production in HEp-2 cells. Superoxide production by complex I is supposed to occur during the reverse electron transport (RET) from ubiquinol to NAD^+^ and during the forward electron transport (FET) from NADH to ubiquinone, the former being faster than the latter. Recent observations have led to the conclusion that rotenone enhances ROS formation during the FET and inhibits it during the RET [19]. Moreover, the RET-induced ROS production is regulated by Δ*ψ*. Generation of O_2_^∙−^ and H_2_O_2_ by the electron transport chain is amplified by an increase in Δ*ψ* [25]. The inhibition of electron transport by antimycin A in complex III resulted in ROS formation even after rotenone treatment (Figure 4). These results indicate that ROS formation by mitochondria in HEp-2 cells under physiological conditions is not connected with the RET in complex I. The RET is observed under conditions of high Δ*ψ* [19]. Previously we have shown that ascorbate at high concentrations induces the decrease in intracellular pH value that can lead to the increase in Δ*ψ* [16]. In such conditions, rotenone inhibits ROS production in mitochondria. Therefore, rotenone decreased ascorbate-induced Ca^2+^ release (Figure 1) and antimycin A-induced Ca^2+^ release (Figure 3). The results obtained indicate that antimycin A activates ROS production by complex III leading to the increase in ascorbate-induced calcium response of HEp-2 cells. Rotenone, in turn, decreases the rise in [Ca^2+^]_cyt_ caused by the action of ascorbate and antimycin A as it blocks electron transfer in complex I of the mitochondrial respiratory chain and inhibits the H_2_O_2_ production in complex III. Decrease in the intracellular pH value enhances oxidative processes in cells [26]; therefore after the ascorbate treatment, the increase in [Ca^2+^]_cyt_ caused by antimycin A occurred even at low concentrations of the inhibitor (Figure 3). Taken together, our results suggest that ascorbic acid can regulate Ca^2+^ release in HEp-2 cells by locally induced ROS production.

The data of this preliminary study point out that complex III may be a possible player of ROS production in mitochondria under ascorbate treatment. It is important to emphasize that multiple sources of ROS generation have been identified in mitochondria [27, 28]. Recently, proapoptotic protein p66^Shc^ has been shown to localize in the mitochondrial intermembrane space and redox cycle with cytochrome c to produce H_2_O_2_ that induces permeability transition [29]. In conditions of rise of ascorbic acid concentration, activation of p66^Shc^ pathways may in part explain enhancement of ROS generation. Further investigation will improve our understanding of mechanisms by which mitochondria are stimulated to produce oxidants and what the proximate targets of such oxidants are. 

**Figure 5:**
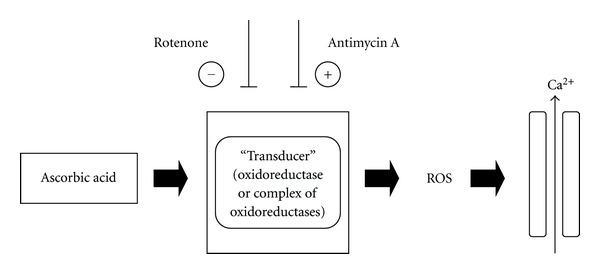
A schematic representation of the mechanism of ascorbate-induced redox regulation of Ca^2+^ release. Signal transduction form redox-active molecule to target occurs with additional participants—oxidoreductases (in our case—mitochondrial enzymes). Mitochondria-derived ROS induce Ca^2+^ release. Signal decoding in cells depends on the transducer activity of additional participant. Antimycin A may increase the ROS production and Ca^2+^ response in cells, while rotenone decreases it.

On the other hand, the results obtained indicate a new possible way of redox regulation of Ca^2+^ signaling (Figure 5). Calcium signaling occurs when the cell is stimulated to release Ca^2+^ from intracellular stores. According to our data, the antioxidant ascorbic acid can regulate Ca^2+^ release by mitochondria-derived ROS. Ascorbate-induced changes of intracellular redox state can be transduced in increase of ROS concentration by components of electron transport chain. Thus in the proposed mechanism of regulation in addition to redox-active molecules and their targets, an additional participant of signal transduction—oxidoreductases (components of electron transport chain)—appears. The important implication of mitochondrial enzymes in redox regulation is that in this case signal decoding in cells depends also on the “transducer” activity in different types of cells. Thus the redox modulation of Ca^2+^ sparks occurs in species- and tissue-specific fashion. Some agents (antimycin A) may increase the ROS production and Ca^2+^ response in cells, while others (rotenone) decrease it (see Figure 5). Local ROS production modifies the amplitude of Ca^2+^ sparks that are sensed and decoded into defined cell actions by a broad variety of cellular effectors.

In general, results of our research are in agreement with the concept of redox signaling that explains oxidant action by compartmentalization of ROS production and oxidant targets [30]. Our observations lead to the conclusion that the key condition of ascorbate cytotoxicity is ROS generation by mitochondria. In these conditions, Ca^2+^ release proceeds as a result of local effects of mitochondrial oxidants. 

Earlier was supposed that selectivity of ascorbate effect on cancer cells was mediated by an altered acid-base balance in tumor tissues [16]. Changes in the activity of electron transport chain components observed in many cancer cells including carcinoma cells [31] may also promote cytotoxic action of ascorbate towards cancer cells. Ascorbic acid, capable of regulating both acid-base and redox states of cancer cells, may serve as a prototype for the development of new anticancer agents with the mechanism of binary regulatory action. Therefore, a detailed understanding of the mechanism of ascorbate-induced ROS production could aid in the development of new anticancer strategies.

## Abbreviations

Δ*ψ*: Transmembrane electric potential difference
DCF: 2′,7′-Dichlorofluorescein
H_2_DCF: 2′,7′-Dichlorodihydrofluorescein
H_2_DCF-DA: 2′,7′-Dichlorodihydrofluorescein diacetate
FET: Forward electron transport
KRH: Krebs-Ringer-HEPES buffer
RET: Reverse electron transport
ROS: Reactive oxygen species.
